# Realising the potential of home blood pressure monitoring in the community: should HBPM be the default?

**DOI:** 10.3399/bjgp22X719441

**Published:** 2022-04-29

**Authors:** Samuel Finnikin, James P Sheppard

**Affiliations:** Institute of Applied Health Research, University of Birmingham, Birmingham; national clinical specialist advisor, Personalised Care Group, NHS England and NHS Improvement.; Nuffield Department of Primary Care Health Sciences, University of Oxford, Oxford.

## BACKGROUND

Helping control patients’ blood pressure is one of the most important things GPs do to reduce premature mortality and morbidity. Home blood pressure monitoring (HBPM) is increasingly used to achieve this vital primary care task.^1^ As recovery from the COVID-19 pandemic continues, there is a renewed focus on identifying patients with undiagnosed hypertension and ensuring that patients with existing hypertension have every opportunity to optimise their blood pressure. Given the increased trend towards remote consulting over the past 18 months, now is an ideal time to ensure that GPs and patients are getting the most out of this simple and effective tool.

While ambulatory blood pressure monitoring (ABPM) remains the gold standard for the diagnosis of hypertension, HBPM may be a reasonable alternative^2^ and is recommended when ABPM is not available or tolerated.^3^ It does, however, carry a risk of overdiagnosis.^4^ HBPM is the most acceptable method of monitoring blood pressure to patients,^5^ particularly among some minority ethnic groups.^6^ However, HBPM is not currently recommended by the National Institute for Health and Care Excellence for monitoring patients with hypertension, but there is good evidence of improved blood pressure control when HBPM is combined with co-interventions such as tailored pharmacist or physician support,^7^ and there are several advantages to patients and practices (Box 1). In practice, HBPM is often more convenient for patients and less resource intensive for practices so is an excellent option for monitoring blood pressure control.

**Box 1.** Pros and cons of community home blood pressure monitoring

**Advantages**	**Disadvantages**
Inexpensive for healthcare providers	Some cost to patients; may contribute to health inequalities
Improved accuracy when compared with office readings	Not as accurate as ambulatory blood pressure monitoring
Engages patients with self-management of their hypertension	Not all patients can do it (or want to)
May improve medicine adherence^8^	Onus on patients to take and report readings
Ideal for remote consultations	Monitors need to be calibrated every few years and may become inaccurate without patient or clinician being aware

## WHAT TO DISCUSS WITH PATIENTS?

For patients who are willing and able to undertake HBPM, it is worth investing some time discussing the use of home monitors to ensure that the readings obtained are accurate. This information sharing could be delegated to other members of the practice team and there are various online resources that can support the process (Box 2).

**Box 2.** Useful resources to support home blood pressure monitoring

British and Irish Hypertension Society (BIHS) validated monitors: https://bihsoc.org/bp-monitors/for-home-use/ BIHS ‘how-to’ guides for patients and practices: https://bihsoc.org/resources/bp-measurement/hbpm/ Academic Health Science Network (AHSN) North East and North Cumbria resources: https://www.ahsn-nenc.org.uk/what-we-do/improving-population-health/cardiovascular-disease-prevention/blood-pressure-monitoring/home-blood-pressure-monitoring-resources-page/ AHSN video for patients: https://www.ahsn-nenc.org.uk/what-we-do/improving-population-health/cardiovascular-disease-prevention/blood-pressure-monitoring/home-blood-pressure-monitoring-resources-page/ British Heart Foundation resources for patients: https://www.bhf.org.uk/informationsupport/support/manage-your-blood-pressure-at-home

If patients are buying a monitor, GPs should advise them to invest in one with an upper arm cuff that has been clinically validated and ideally is recommended by the British and Irish Hypertension Society. More expensive machines add features but do not increase accuracy, so the cheapest machines (usually <£20) will suffice. Patients with particularly large or small upper arms may need to purchase an appropriately sized cuff. If the patient already has a monitor, or is borrowing one, the clinician should mention that, if it is >5 years old, it may be inaccurate or require calibration to ensure it is reliable. The cost of obtaining a monitor may be a barrier; to support patients, some practices have a scheme to lend out monitors. There may also be local or national schemes to provide free blood pressure monitors to patients who would benefit the most.

When taking readings, patients should be told to sit comfortably, with their feet flat on the floor, for 5 min prior to taking their blood pressure. Ideally, they should avoid caffeine or alcohol for at least 2 hours prior to taking their blood pressure. Two readings, taken at least a minute apart, are preferable; with the patient recording both. For diagnosis, at least 5 days of readings should be used, with the average being taken (excluding the first day of readings.) For monitoring purposes, it may be pragmatic to be more flexible. Even one reading gives an idea of control and is correlated with cardiovascular disease (CVD) risk,^9^ but a greater number will increase accuracy and better inform decision making.

Finally, if there are concerns that home readings being submitted may be inaccurate, then the GP should consider inviting the patient for a review. At this review, the GP should ensure the patient is using the correct technique for taking readings, check that the machine they are using is not old or damaged, and perhaps take a clinic reading for comparison (although it must be remembered that clinic readings may not be more accurate than home readings).

## WHAT TO DO WITH THE READINGS?

It is important that the patient understands what their blood pressure numbers mean. A target to aim for should be agreed upon. It should be remembered that HBPM targets are lower than clinic readings. For most people with hypertension, a target of <135/85 mmHg is appropriate, but this should be adjusted to <145/85 mmHg for adults aged ≥80 years.^3^ Patients may worry and seek urgent advice should their BP be much higher than this. It is a good idea for the GP to pre-empt these situations by explaining that high one-off readings are seldom concerning, but recommend they contact the GP if their BP is persistently over 170/115 mmHg.

The frequency of readings should also be agreed, although there is little evidence to inform this decision. It is unnecessarily burdensome for patients to take their blood pressure every day. A GP may only want the results sent to them once a year, but could suggest the patient takes readings more frequently, only letting the GP know if their readings are above their target. Every patient is different. Personalising monitoring can reduce the burden of hypertension and increase efficiency of care.

Several ways exist for patients to let GPs know their HBPM readings. It should be considered what method is preferable for the patient as well as the ease of processing by the practice team; a paper form may be easiest for some patients, but requires manual processing; an online form or SMS-based system, though requiring some technological literacy, may be preferable since it may automatically calculate and code average results. SNOMED codes for average home systolic and diastolic blood pressure should be used.

If blood pressure is not below target (for example, 135/85 mmHg) then a discussion about further options to reduce blood pressure should be arranged. In some circumstances, pre-agreed actions could be put into place (for example, up-titrating an antihypertensive and rechecking). It is also worth taking note of the lowest and highest readings. Some patients have particularly variable blood pressures. If GPs only focus on the average, they risk dropping their lowest readings too low and causing symptomatic and problematic hypotension or other harms from overtreatment.^10^

## CONCLUSION

HBPM is a fundamental component of the management of hypertension and there is a good argument to say that using clinic readings should be the exception, rather than the norm, in modern primary care. Encouraging patients to incorporate HBPM as part of their self-management, and having clear systems to communicate and respond to results, can optimise efficiency and effectiveness of hypertension management in primary care.
